# Mitigating Salt Stress with Biochar: Effects on Yield and Quality of Dwarf Tomato Irrigated with Brackish Water

**DOI:** 10.3390/plants13192801

**Published:** 2024-10-06

**Authors:** Matteo Lentini, Michele Ciriello, Youssef Rouphael, Petronia Carillo, Giovanna Marta Fusco, Letizia Pagliaro, Francesco Primo Vaccari, Stefania De Pascale

**Affiliations:** 1 Department of Agricultural Sciences, University of Naples Federico II, 80055 Portici, Italy; matteo.lentini@unina.it (M.L.); michele.ciriello@unina.it (M.C.); 2 Department of Environmental, Biological and Pharmaceutical Sciences and Technologies, University of Campania “Luigi Vanvitelli”, Via Vivaldi 43, 81100 Caserta, Italy; petronia.carillo@unicampania.it (P.C.); giovannamarta.fusco@unicampania.it (G.M.F.); letizia.pagliaro@unicampania.it (L.P.); 3 Institute of BioEconomy—Biology, Agriculture and Food Sciences Department, National Research Council of Italy, Via Caproni 8, 50144 Firenze, Italy; francesco.vaccari@ibe.cnr.it

**Keywords:** abiotic stresses, amendment, horticultural, lycopene, *Solanum lycopersicum* L.

## Abstract

The increase in the frequency and magnitude of environmental stresses poses a significant risk to the stability of food supplies. In coastal areas of the Mediterranean, brackish water has long been considered a limitation on horticultural production. In this scenario, the use of biochar in agriculture could be considered a valuable tool to cope with the deleterious effects of salt stress. This work aimed to investigate, in a protected environment, the effects of different concentrations of biochar (0, 1, and 2% *v*/*v*) obtained from poplar (*Populus* L.) biomass on the yield and quality of dwarf San Marzano ecotype tomatoes irrigated with saline water at different concentrations of NaCl (0, 40 and 80 mM). The increase in salt concentration from 0 to 80 mM NaCl reduced the total yield (−63%) and the number of fruits (−25%), but improved the main quality parameters such as dry matter (+75%), total soluble solids (+56%), and polyphenol content (+43%). Compared to control conditions, biochar supplementation improved the total yield (+23%) and number of fruits (+26%) without altering the functional and organoleptic characteristics of the fruits. The promising results underscore the potential of biochar as a sustainable solution to amend soils in order to improve tomato production under unfavorable conditions such as high salinity. However, there is a need to clarify which adaptation mechanisms triggered by biochar amending improve production responses even and especially under suboptimal growing conditions.

## 1. Introduction

In recent years, increasing world population, urbanization, and rapid industrialization have necessarily increased resource use [1]. Therefore, the depletion of natural resources is now inevitable. Biomass can be a potential substitute for raw materials for environmental conservation [2]. Biomass consists of organic plant material, such as agri-food wastes, with high physicochemical and biological potential, which can be converted to biochar through pyrolysis [3]. The conversion in biochar of these residual biomasses has gained popularity for the possibility to reduce atmospheric CO_2_ emissions while fostering their environmental applications with a circular economy approach [4]. Over the past few years, the production and use of biochar have increasingly gained the attention of the scientific research community [5,6,7]. Biochar is a carbon-based material obtained through thermal degradation (pyrolysis) of organic biomasses under hypoxic conditions [8]. Generally, the most used biomasses for biochar production are agricultural products, forest debris, bioenergy crops, sewage sludge, and animal wastes [9]. Residual biomasses undergo pyrolysis, a thermal degradation process performed at temperatures ranging from 300 °C to 1000 °C, in which they are converted into a stable and recalcitrant form of organic carbon [10]. The physical and chemical characteristics of biochar depend on the conditions of the pyrolysis process, especially the temperature and oxygen availability, as well as the type of biomass used [11]. A growing number of scientific works have highlighted the positive role of biochar soil amendment, especially in adverse agricultural contexts [12,13,14]. The main challenge for the agricultural world is to feed an ever-increasing human population in the face of increasing yield losses due to climate change [15].

Among the various abiotic stresses that limit quanti–qualitative production, NaCl salt stress is one of the most prevalent and common ones, particularly affecting coastal areas [16]. Moreover, high salinity in arid and semi-arid regions is a major challenge for agricultural production, food security, and sustainability, as it negatively impacts plant growth [17]. In addition, the increased concentration of toxic ions (Na^+^ and Cl^−^) in the rhizosphere triggers physiological alterations and metabolic disturbance. In particular, the decrease in water potential due to salts in the soil makes it difficult for plants to uptake water and mineral ions. The reduction in water availability affects plant transpiration, limiting photosynthetic efficiency and photosynthate production, which inevitably negatively impacts on plant growth rate and overall production [18,19,20]. Recent studies highlight how the exogenous application of biochar obtained from wheat straw and stem wood was able to mitigate salt stress on sorghum (*Sorghum bicolor* L.) and chickpea (*Cicer arietinum* L.) plants [21,22]. Specifically, it has been shown that biochar supplementation in salinized soils would improve water-retention capacity by reducing osmotic and oxidative stresses related to the presence of Na^+^ and Cl^−^ in the circulating solution [23]. In addition, soil amendment with biochar would promote cation exchange capacity and hydraulic conductivity [24], thereby increasing macronutrient acquisition and carbon fixation capacity, ultimately promoting plant growth [25]. The biochar’s high adsorption capacity can mitigate the detrimental impact of salinity by lowering its availability in soil [26]. This reduction in Na^+^ limits its uptake and accumulation in plant tissues, resulting in lower electrolyte loss even at higher salinity levels [27]. In similar saline contexts, observed increased enzymatic antioxidant activity following biochar supplementation [22,28]. This enhanced activity boosts the plants’ response to suboptimal conditions in common bean (*Phaseolus vulgaris* L.) and chickpea plants. In addition to improving the physicochemical characteristics of soils partially compromised by high salinity, several studies have shown that the supplementation of biochar could improve, regardless of the presence of NaCl, the germination rate, and quali–quantitative yield of different vegetables [8,29,30]. In this view, the addition of an appropriate amount of biochar can increase the yield of tomatoes [31,32,33], as well as the amount of total soluble solids and lycopene content [34]. Tomato (*Solanum lycopersicum* L.) represents one of the most significant and cultivated horticultural crops worldwide. Tomatoes are grown both in protected crops and in open fields. However, creating a more sustainable production system is essential to improve crop growth and achieve high yields. Economic sustainability principles are one of the main reasons behind greenhouse tomato production [35].

Tomato fruits are widely consumed as fresh and/or processed food products [36,37]. These are characterized by a high content of nutrients and bioactive substances (e.g., fiber, organic acids, minerals, phenolic compounds, and carotenoids) directly related to numerous human health benefits [38,39]. In the field of plant research, tomato has long been a key model organism for studying fruit development, genetic traits, and stress tolerance [40]. Tomato, originally from western South America, had wild ancestors that were well suited to the salinity of coastal regions. However, the domestication process has produced modern cultivars losing their natural salt tolerance [41,42]. The hypothesis of our research was to take advantage of biochar soil amendment to mitigate the deleterious effects related to soil salinization. This study aimed at to examine how the varying concentration of biochar (0, 1, and 2% *v*/*v*) impacts the yield and quality of a dwarf tomato San Marzano ecotype irrigated with brackish water at different salinity levels (0, 40, and 80 mM NaCl).

## 2. Results and Discussion

### 2.1. Yield Response of Tomato to Salinity and Biochar Application

**Figure 1:**
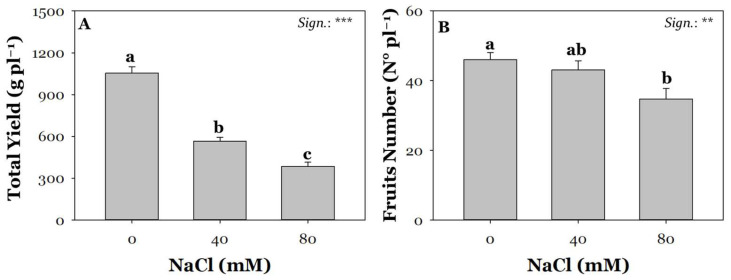
Effect of saline irrigation with three NaCl levels (0, 40, 80 mM) on total yield (**A**) and fruit number (**B**). Mean comparisons were performed by Tukey’s HSD post-hoc test. Different letters within each column indicate significant differences between means (*p* ≤ 0.05). ** and *** denote significant effects at *p* ≤ 0.01 and *p* ≤ 0.001, respectively (*n* = 3).

Tomatoes are among the most consumed vegetables worldwide and are a key crop for the economies of many countries [43,44]. Global tomato production is approximately 180 million tons. The leading producers include China, with around 63 million tons, India, with 19 million tons, Turkey, with 13 million tons, the USA, with 11 million tons, Egypt, with 7 million tons, and Italy, with 5 million tons [45]. However, potential tomato yields are increasingly threatened by escalating biotic and abiotic pressure. NaCl salinity strongly influences all vital aspects of the plants, affecting morphological and physiological characteristics [46]. Specifically, continuous exposure of tomato plants to NaCl salinity causes negative effects on growth and production performance [47]. Today, to contrast the deleterious effects of salinity, biochar soil amendment appears to be a promising and interesting agronomic practice also from an environmental sustainability perspective [48,49]. In agreement with a similar study by Zhang et al. [50], total yield decreased significantly with increasing NaCl concentration (0 mM > 40 mM > 80 mM; Figure 1A). Compared with control conditions, plants exposed to the highest NaCl concentration (80 mM) had a 63% decrease in yield (Figure 1A). Fruit yield was strongly affected by the exposure to 80 mM NaCl salinity, probably due to the lower water potential, which reduced the plant water uptake capacity and consequently the availability of macronutrients needed for growth. In fact, increased NaCl concentration leads to drastic hyperosmotic stress, nutrient imbalance, and ionic toxicity that impair plant cellular functions and cause the production of reactive oxygen species (ROS) [51,52]. ROS are initially produced at electron transport chain levels as a retrograde signal for communicating dysfunctional events to the nucleus. However, when their production further increases, since they are highly reactive and can trigger lipid peroxidation, damaging membranes, enzymes, proteins, and nucleic acids [53]. The decrease in yield is partially evidenced by a notable decline in the number of fruits per plant (Figure 1B), as also observed by Massaretto et al. [54]. Compared with non-saline conditions, the number of fruits from plants salinized at 80 mM NaCl was 24% lower.

**Figure 2:**
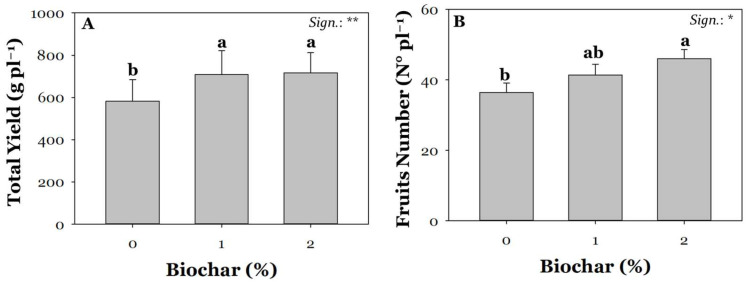
Effect of biochar treatment at three different concentrations (0, 1, and 2%) on total yield (**A**) and fruit number (**B**). Mean comparisons were performed by Tukey’s HSD post-hoc test. Different letters within each column indicate significant differences between means (*p* ≤ 0.05). * and ** denote significant effects at *p* ≤ 0.05 and *p* ≤ 0.01, respectively (*n* = 3).

The strong decrease in the number of tomato fruits, particularly under the highest salinity treatment of 80 mM NaCl, is likely due to an increased rate of flower abortion. This phenomenon is caused by reduced pollen viability and lower fertilization success in plants exposed to severe salinity [55]. In contrast, the number of fruits produced by plants under mild salinity (40 mM NaCl) did not differ from the control. The yield loss observed under 40 mM NaCl treatment (−46%) is likely due to a lower average fruit weight rather than a reduction in the total number of fruits. Salt stress effects could be mitigated by using various agronomic strategies, such as the targeted management of irrigation and fertilization practices, the use of biostimulants, and soil amendments [16]. Regardless of the average salinity (S) effect, biochar supplementation resulted in a significant increase (23% on average) in total yield (Figure 2A). This finding aligns with numerous studies that have consistently reported positive outcomes on tomatoes when supplemented with biochar. The literature review suggests several mechanisms that may be responsible for the beneficial effects of biochar on crop growth and yield. Some of these are mainly related to alterations of physicochemical and biological changes in the soil [56,57], while others are related to mechanisms of immobilization of essential macro- and micronutrients in the soil [58]. Like what was observed for yield, the number of fruits per plant increased by 26% compared to the control, especially when biochar was added at 2% (Figure 2B).

Many studies have shown that biochar increases soil mineral content due to its high cation exchange capacity. In fact, the improved production performance of tomato plants could be a direct consequence of the biochar-induced increased assimilation of phosphorus, nitrogen, and potassium, also due to positive action on the soil microbiome [59,60,61].

### 2.2. Effect of Salinity and Biochar Application on Visual, Organoleptic, and Nutritional Quality

**Figure 3:**
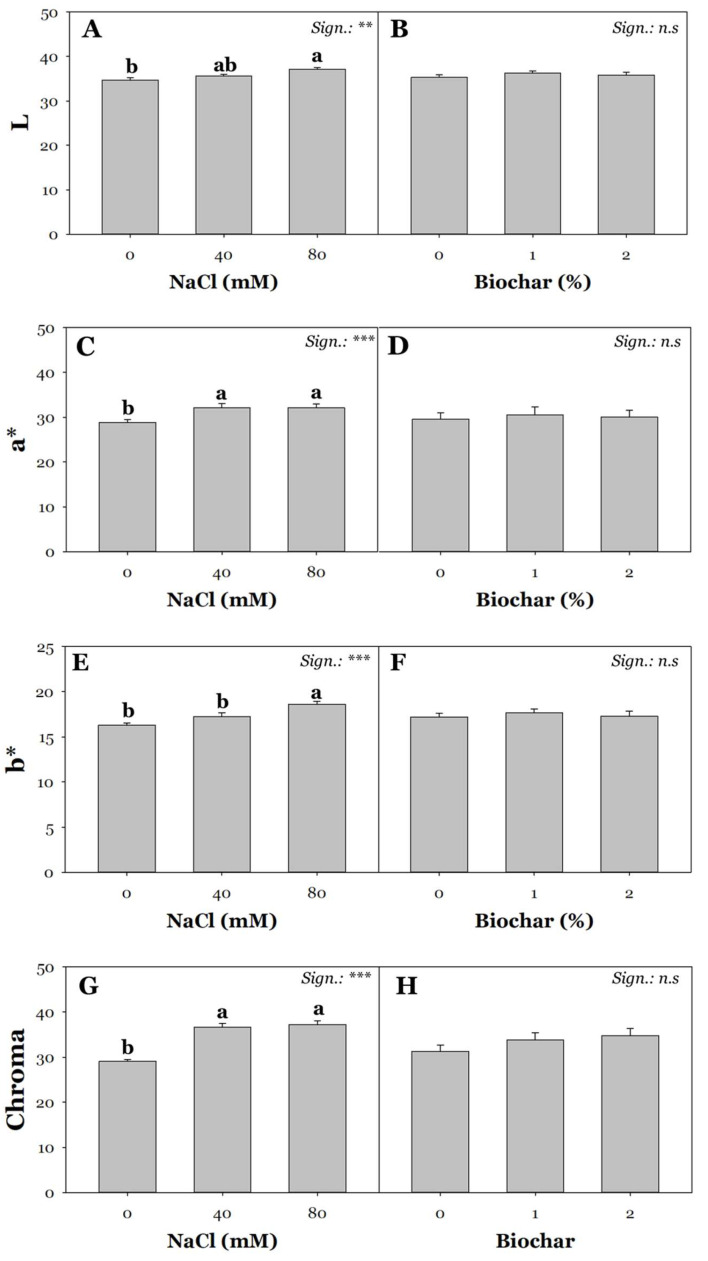
Effect of saline irrigation with three NaCl levels (0, 40, 80 mM) and effect of biochar treatment at three different concentrations (0, 1, and 2%) on CIELab color space parameters: L (**A**,**B**), a* (**C**,**D**), b* (**E**,**F**), and chroma (**G**,**H**). Mean comparisons were performed by Tukey’s HSD post-hoc test. Different letters within each column indicate significant differences between means (*p* ≤ 0.05). ** and *** denote significant effects at *p* ≤ 0.01 and *p* ≤ 0.001, respectively (*n* = 3).

An increasing interest in superior agri-food products has driven growers to meet the changing needs of increasingly discerning consumers. In the past, vegetables were selected based on aesthetic characteristics; nowadays, organoleptic and sensory characteristics are leading parameters guiding the choice of end consumers [62,63]. The organoleptic quality of tomato fruits is primarily influenced by the levels of soluble solids, including glucose, fructose, and sucrose. These sugars, together with organic acids and amino acids, comprise approximately 75% of the fruit’s dry matter. Unlike the yield parameters described above (Figure 1A,B and Figure 2A,B), CIELab colorimetric analyses (L, a*, b*, Chroma) showed significant differences due solely to salinity treatment (Figure 3). Specifically, plants subjected to high NaCl concentrations (80 mM) showed L (+7%), a* (+34%), and b* (+14%) values compared to those present in control conditions. Regardless of the salt level (40–80 mM), salinized fruits exhibited higher values of chroma (on average by 13%) (Figure 3G), which, as suggested by Formisano et al. [64], would indicate a higher intensity of color perceivable by the human eye. Accordingly, Espley and Jaakola [65] found that salinity caused an increase in perceivable color due to changes in a* and b* values.

As reported in Table 1, both parameters (dry matter and TSS) were found to be significantly affected by the mean effect S. Specifically, regardless of NaCl level, the salinization of the circulating solution led to an average increase in dry matter by 75% and TSS by 56%, compared to control conditions, confirming the findings of Agius et al. [66] on tomato plants.

It has been suggested that the higher TSS values recorded in fruits of plants salinized at 40 and 80 mM are probably a consequence of the lower ability to translocate water, a hypothesis that is confirmed by the higher dry matter contents (Table 1) recorded in fruits of salinized plants. The absence of fertilizer supplementation in the experimental trial likely reduced the production of nitrogen-containing compounds. This shortage caused a decrease in the necessary nitrogen-based osmolytes required to osmotically balance the toxic ions compartmentalized in the vacuole with those in the cytosol. Consequently, there was an accumulation of soluble sugars not utilised to produce amino acids or nitrogen-based osmolytes. However, these soluble sugars can still exert a function as osmolytes [67]. The higher dry matter values could extend the shelf-life of the product. This result highlights how biochar amendment in addition to resulting in increased yield would also improve aspects related to fruit organoleptic quality, confirming the results reported by [68] on tomato. As previously described, biochar application may improve soil properties, promoting root development, and in particular root length, projected root area, and surface area, as well as the number of root forks and crossings, enhancing water and nutrient uptake under a stressed environment. Consequently, this boosts photosynthates production, which can be allocated to developing fruits [60,69].

In contrast, pH values (Table 1) (average 4.23) were not significantly affected by the average S effect, unlike what was observed in a similar study on tomatoes (cv *Gustafano*) subjected to different salt treatments (17 mM and 34 mM NaCl) [66]. Just the lower levels of NaCl tested by Agius, von Tucher, and Rozhon [66], as well as the different growth system (hydroponics) used, could justify the different results obtained in our study. Even though, in the literature, higher EC values of juice are often positively correlated with TSS values [70], the results of our study only partially confirm this. Compared with control conditions, fruit from plants under moderate salinity (40 mM) were characterized by higher TSS (+50%) but not equally high EC values (Table 1). On the contrary, under severe salinity conditions (80 mM), the fruits showed higher EC values than the control, and moderate salinity conditions (40 mM). The fruits probably obtained under severe salinity (80 mM) had not only higher TSS but also differences in the content of organic acids, recognized compounds capable of altering the EC of tomato juice [71].

**Figure 4:**
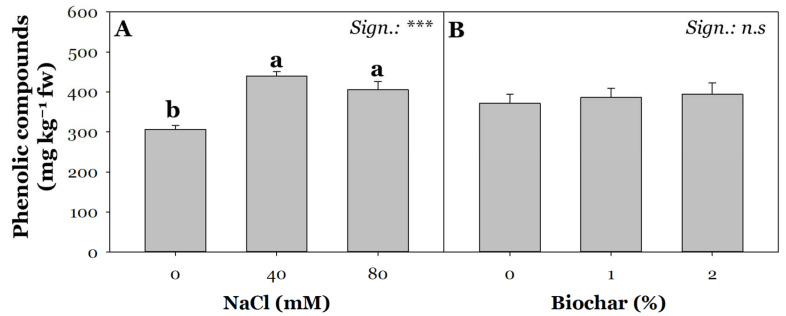
Effect of saline irrigation (**A**) with three NaCl levels (0, 40, and 80 mM) and biochar treatment (**B**) at three different concentrations (0, 1, and 2%) on phenolic compounds. Mean comparisons were performed by Tukey’s HSD post-hoc test. Different letters within each column indicate significant differences between means (*p* ≤ 0.05). n.s. and *** denote non-significant and significant effects at *p* ≤ 0.001, respectively (*n* = 3).

A high concentration of NaCl in the circulating soil solution generally stimulates the plant defence system, thus leading to increased biosynthesis and accumulation of secondary metabolites in different plant tissues [72]. Phenolic compounds play a key role in plant development and growth regulation, adaptation to the environment, and increased plant tolerance to stressful conditions [73]. A common characteristic of plant secondary metabolites is their ROS scavenging activity [74]. Owing to their well-known antioxidant properties, these bioactive metabolites are considered major players in reducing the incidence of cardiovascular disease in humans [75,76]. The concentration of phenolic compounds in tomatoes is influenced by several aspects, including the plant’s genotype, the cultivation method used, water availability, and salinity [77]. Although it is widely discussed in the literature that the effects of salinity on polyphenol content highly depend on genotype and salt level, our results show an increase in polyphenol content as early as 40 mM NaCl (43%) and 80 mM (32%) (Figure 4A) [78]. The increased biosynthesis of antioxidant compounds recorded in response to salt stress conditions represents a defensive mechanism aimed at decreasing the harmful effects induced by NaCl [79].

Regardless of salt treatment, biochar application did not result in significant differences in polyphenol content (Figure 4B), different from that recorded on tomatoes (cv Rio Grande) by Petruccelli et al. [80]. However, as argued by the same authors, beneficial effects of biochar on plant secondary metabolism are strongly influenced by the different physical–chemical characteristics of biochar, application dose, and growth conditions.

**Figure 5:**
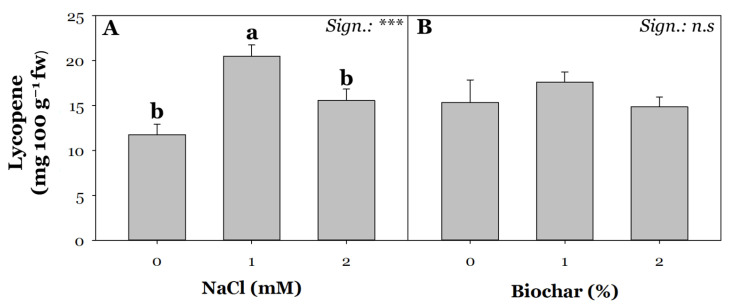
Effect of saline irrigation (**A**) with three NaCl levels (0, 40 and 80 mM) and biochar treatment (**B**) at three different concentrations (0, 1, and 2%) on lycopene content. Mean comparisons were performed by Tukey’s HSD post-hoc test. Different letters within each column indicate significant differences between means (*p* ≤ 0.05). n.s and *** denote non-significant and significant effects at *p* ≤ 0.001, respectively (*n* = 3).

Similar to the observations for polyphenols (Figure 4A,B), lycopene content was also found to be significantly affected only by the average S effect (Figure 5A,B). Lycopene metabolism can be modulated by water deficit [81], low light radiation [82], and salt stress [83]. Lycopene has a well-known biological activity. Several studies reported in the critical review by Kulawik et al. [84], in addition to providing valuable insights into the mechanism of action of this carotenoid, demonstrate its potential usefulness in individuals with cardiovascular problems, nervous system disorders, and liver-related diseases. The positive effect of lycopene on human health is a result of its pleiotropic effect.

In agreement with De Pascale et al. [85] and Kubota et al. [86], the imposition of moderate salt stress (40 mM) increased lycopene content compared with non-salinized conditions by 74% (Figure 5A). Moreover, several authors observed a drastic reduction in lycopene content in salinized tomato fruits due to reduced uptake of K^+^ ion, which acts as a cofactor for numerous enzymes, playing a crucial role in the biosynthesis of isopentenyl diphosphate, the initial precursor in the mevalonate pathway for carotenoid production [87]. It is important to note that our results (Figure 4 and Figure 5) were expressed on a fresh base and not on a dry base, as the previous authors cited above reported. Consequently, the increase in dry matter (Table 1) certainly played a key role. In any case, the significant reduction of lycopene recorded in tomato fruits under severe salinity compared with those at moderate salinity would partly confirm the negative impact of high salinity on lycopene biosynthesis and accumulation.

## 3. Materials and Methods

### 3.1. Experimental Site, Plant Material, and Experimental Design

The experimental trial, aimed at assessing the effects of biochar supplementation on tomato (*Solanum lycopersicum* L.) plants irrigated with different NaCl levels, was conducted in an unheated greenhouse at the Department of Agriculture (DIA), University of Naples Federico II, in Portici (Naples, latitude 40°49′11″ N, longitude 14°20′28″ E, 29 m above sea level). Tomato seeds (ecotype “Dwarf San Marzano”) purchased from Semiorto Seeds (Pagani, Salerno, Italy) were germinated on peat in 0.25 m^2^ polystyrene panels. At 3rd–4th true leaf stage, the plants were transplanted into 5 L pots (diameter 19), with a planting density of 11 plants m^−2^. The chemical and physical characteristics of the soil used are shown in Supplementary Table S1. Biochar was obtained by gasification of wood chips derived from poplar (*Populus* sp.) (Supplementary Table S2), supplied by the company “CMD Engine”, located in Caserta, Italy. The biochar was supplemented with three different percentages (0, 1, 2%, *v*/*v*).

Salinity was set at 14 days after transplanting at different concentrations: 0, 40, 80 mM NaCl; osmotic water was used for irrigation, and the experimental trial did not include fertilizer supplementation. The experiment utilized a randomized block design with a factorial arrangement of three salinity levels (0, 40, and 80 mM) and three increasing percentages of biochar (0, 1, and 2% *v*/*v*), with each treatment replicated three times. Each replication consisted of six plants. The experiment had a total duration of 84 days.

### 3.2. Fruit Harvest, Yield, and Fruit Quality Measurement

At the end of the experiment (28 June), the total yield and number of fruits were determined for each plant. A fraction of the harvested fruit was blended with a 2 L capacity Waring^®^ blender (HGB140, McConnellsburg, PA, USA) for 60 s to determine the total soluble solids (TSS) content (Atago Co., Ltd., Tokyo, Japan). Using the same juice, pH and electrical conductivity (EC) values were measured using a digital pH meter (model HI-9023; Hanna Instruments, Padua, Italy). Finally, the dry matter percentage (DM% = dry weight/fresh weight ×100) was determined by an aliquot of juice (about 100 g), which was dried at 70 °C until reaching constant weight. A portion of the fruit was shock-frozen in liquid nitrogen at harvest and subsequently stored at −80 °C for later quality analysis.

### 3.3. Determination of Colorimetric Parameters

Twenty-five fruits per replicate were selected for the determination of CIELab colorimetric indices (L, a*, and b*). Using a Minolta Chromameter CR-400 handheld colorimeter (Minolta Camera Co., Ltd., Osaka, Japan), two measurements were made for each fruit. Chroma values were calculated according to the following formula:

$$Chroma=\sqrt{a^{2}+b^{2}}$$

### 3.4. Quality Parameters

For phytochemical analysis, representative samples of frozen tomato fruits were freeze-dried and then finely ground. Analyses for the determination of total phenols and lycopene were performed by spectrophotometry (Hach DR 4000, Hach Co., Loveland, CO, USA) according to the method proposed by Vl [88] and Sadler et al. [89], respectively. Pure gallic acid and lycopene standards, purchased from Sigma Aldrich (Milan, Italy), were used to develop calibration curves for the quantification of total phenols and lycopene amounts. To determine total phenols and lycopene levels, UV-VIS spectrophotometry was employed at absorbances of 765 and 472 nm, respectively.

### 3.5. Statistics Analysis

Statistical analysis was performed using IBM SPSS Statistics software (SPSS Inc., Chicago, IL, USA) version 26.0 for Windows 10. A two-way analysis of variance (ANOVA) was conducted to evaluate the significance of the effects and interactions between salt stress (S) and biochar factor (B). In addition, a one-way ANOVA was applied to compare the average effects of S and B, determined using the Tukey–Kramer HSD test, which was also used to determine statistical differences, with a significance level of *p* < 0.05.

## 4. Conclusions

**Figure 6:**
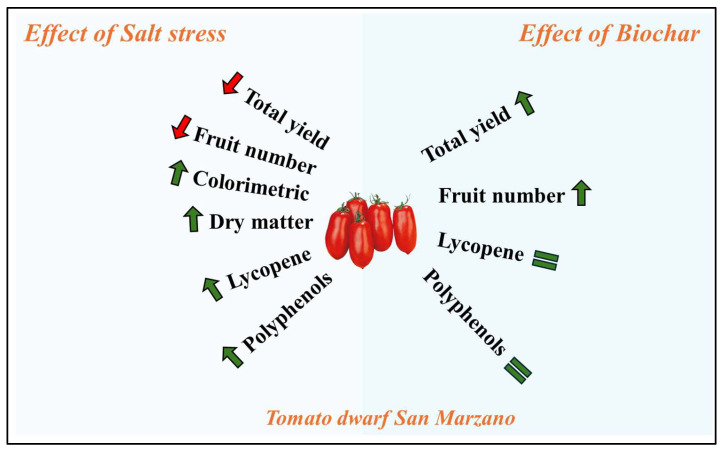
Schematic graphical representation of how the yield and organoleptic properties of varying concentration of biochar (0, 1, and 2% *v*/*v*) impact the yield and quality of a dwarf tomato San Marzano ecotype irrigated with brackish water at different salinity levels (0, 40, and 80 mM NaCl).

The purpose of our study was to investigate the potential of biochar, derived from poplar waste biomass, on the yield and organoleptic and functional properties of a tomato ecotype (dwarf San Marzano) subjected to increasing concentrations of NaCl (0, 40, and 80 mM). Although tomato is generally classified as a moderately salinity-tolerant species, the results revealed the glycophytic behaviour of the ecotype tested, and a significant reduction in total yield, even at low salinity levels (40 mM NaCl). On the other hand, salinization of irrigation water increased fruit quality parameters, such as total polyphenol content, total soluble solids, lycopene, and the percentage of dry matter content. However, the most interesting results were related to biochar amendment (Figure 6). Regardless of its concentration (1 and 2% *v*/*v*), the application of biochar to the soil significantly improved total tomato yield under both control and salt stress conditions. Although our results refer to a short-lived pot experiment, they provide interesting and promising insights into the use of biochar for horticultural crops of economic interest. However, it is crucial to acknowledge that the economic viability of biochar is context-specific. Factors like biochar type, application methods, crop selection, local climate, and market prices all influence the cost–benefit analyses. Furthermore, in situ biochar production as a byproduct of energy production from biomass could enhance the economic viability of biochar application, making it a more attractive option for growers. Thorough research and potentially small-scale trials are recommended before large-scale adoption.

## Figures and Tables

**Table 1:** Effect of Saline irrigation (S) with three NaCl levels (0, 40, and 80 mM) and Biochar treatment (B) at three different concentrations (0, 1, and 2%) on dry matter %, total soluble solids (TSS), juice pH, and EC.

Treatment	Dry Matter	TSS	Juice pH	Juice EC
	%	°Brix		
Saline irrigation (S)				
0	7.33 ± 0.21 b	6.46 ± 0.13 b	4.33 ± 0.02	4.06 ± 0.08 b
40	12.85 ± 0.27 a	9.72 ± 0.20 a	4.11 ± 0.11	4.71 ± 0.38 b
80	12.58 ± 0.58 a	10.09 ± 0.25 a	4.25 ± 0.02	6.38 ± 0.55 a
Sign	***	***	n.s	***
Biochar (B)				
0	10.48 ± 0.94	8.57 ± 0.62	4.29 ± 0.03	5.27 ± 0.45
1	10.92 ± 0.84	8.72 ± 0.53	4.15 ± 0.12	4.65 ± 0.31
2	11.36 ± 1.11	8.98 ± 0.66	4.25 ± 0.02	5.23 ± 0.69
Sign	n.s	n.s	n.s	n.s
S x B	n.s	**	n.s	*

Mean comparisons were performed by Tukey’s HSD post-hoc test. Different letters within each column indicate significant differences between means (*p* ≤ 0.05). ns, *, ** and *** denote non-significant and significant effects at *p* ≤ 0.05, *p* ≤ 0.01, and *p* ≤ 0.001, respectively (*n* = 3).

## Data Availability

The data presented in this study are available on request from the corresponding authors.

## Supplementary Materials

The following supporting information can be downloaded at: https://www.mdpi.com/article/10.3390/plants13192801/s1. Table S1. Physical and chemical properties of soil. Table S2. Physical and chemical properties of biochar.
