# Supplementary material for: Mitigating Salt Stress with Biochar: Effects on Yield and Quality of Dwarf Tomato Irrigated with Brackish Water
Source: Plants (Basel). 2024 Oct 6;13(19):2801. doi: 10.3390/plants13192801 (PMC11478744; doi:10.3390/plants13192801)
Supplement: Supplementary file 1 [file plants-13-02801-s001.zip › plants-3225076-Supplementary Materials.pdf]

plants

MDPI

Table S1. Physical and chemical properties of soil.

Properties	Unit	Results
Sand	%	81.5
Silt		13.50
Clay		5
N		0.30
P	mg kg^{-1}	68
K		968
Organic matter	%	5.17
pH		6.78
EC	dS m^{-1}	0.7

Table S2. Physical and chemical properties of biochar.

Properties	Unit	Results
**C**	g $100g^{-1}$	71.79
**H**		0.76
**N**		0.58
**S**		0
**Ca**	g kg^{-1}	3.06
**P**		2.83
**K**		1.05
**Mg**		0.76
**Na**		0.33
**Zn**	mg kg^{-1}	36.09
**Cr**		21.25
**Cd**		1.37
**pH**		8.2
**EC**	dS m^{-1} at 25 °C	0.38
**Bulk density**	g m^{-3}	0.22
**Mean particle diameter**	mm	<5.0
